# Using Peer Support to Strengthen Mental Health During the COVID-19 Pandemic: A Review

**DOI:** 10.3389/fpsyt.2021.714181

**Published:** 2021-07-12

**Authors:** Rahul Suresh, Armaghan Alam, Zoe Karkossa

**Affiliations:** ^1^Department of Neurology and Neurosurgery, Montreal Neurological Institute, Montreal, QC, Canada; ^2^Peer Support Centre, McGill University, Montreal, QC, Canada; ^3^Department of Medicine, University of British Columbia, Vancouver, BC, Canada

**Keywords:** COVID-19, coronavirus pandemic, mental health, peer support, healthcare workers, isolation, lockdown, distress

## Abstract

**Background:** The coronavirus (COVID-19) pandemic has had a significant impact on society's overall mental health. Measures such as mandated lockdowns and physical distancing have contributed to higher levels of anxiety, depression, and other metrics indicating worsening mental health. Peer support, which is peer-to-peer provided social and emotional support, is an underutilized and effective mental health resource that can potentially be used to ameliorate mental health during these times.

**Objective:** This review aims to summarize the toll that this pandemic has had on society's mental health as found in peer-reviewed literature from October 2019 to March 2021, as well as suggest the utility of peer support to address these needs.

**Methods:** References for this review were chosen through searches of PubMed, Web of Science, and Google Scholar for articles published between October 2019 and March 2021 that used the terms: “coronavirus,” “COVID-19,” “mental health,” “anxiety,” “depression,” “isolation,” “mental health resources,” “peer support,” “online mental health resources,” and “healthcare workers.” Articles resulting from these searches and relevant references cited in those articles were reviewed. Articles published in English, French and Italian were included.

**Results:** This pandemic has ubiquitously worsened the mental health of populations across the world. Peer support has been demonstrated to yield generally positive effects on the mental health of a wide variety of recipients, and it can be provided through numerous accessible mediums.

**Conclusions:** Peer support can overall be beneficial for improving mental health during the COVID-19 pandemic and may be an effective tool should similar events arise in the future, although the presence of a few conflicting studies suggests the need for additional research.

## Introduction

The COVID-19 pandemic has had a devastating impact on society, from the closure of businesses, disrupted services, and a mounting death toll of millions worldwide. Many individuals, even those who were not directly impacted by this virus, were placed into lockdowns instituted by their respective municipalities, states/provinces, and countries. This enforced isolation has resulted in greater rates of depression, anxiety, psychological distress, insomnia, denial, and anger around the world (1, 2). As the mandated isolations continue to become more frequent and longer in duration, the rates of depression, anxiety, and loneliness are only predicted to increase, especially among children, adolescents and young adults (18- to 24-year olds) (3).

Given that prolonged isolation can be quite harmful for individuals when left unaddressed, there needs to be a way to live with implemented public health measures while mitigating their negative effects on one's mental health. One could argue that individuals should be more proactive in consulting professional mental health services before their mental health declines significantly. However, only 36% of American university students who screened positive for major depression and 52% of students that screened positive for generalized anxiety disorder or panic disorder pursued professional mental health services, despite the sample having access to nearly universal health insurance and various free mental health resources (4–6). Furthermore, in Canada, it was found that individuals from ages 15 to 24 are the least likely age group to seek professional aid for their mental health, despite being the most affected by mental illness (7). Thus, the need for individuals to better support one another becomes apparent, much like first aid training among the general populous. A way to accomplish this would be through the provision of peer support from one individual to another, and unlike physical first aid, this does not require any official certification. Peer support is defined as the social and emotional support offered by an individual in equal standing, founded on respect, shared responsibility, and a mutual agreement of what is helpful (8). Due to it being an informal form of support that is widely accessible and effective, it led us to hypothesize that it would be a valuable tool in aiding the mental health of individuals who are negatively affected by the pandemic. This review discusses the mental health implications of the COVID-19 pandemic as found in peer-reviewed literature during October 2020–March 2021, and further, we suggest the usefulness of peer support as a form of mental health support during these trying times.

## Methods

### Database Search for Literature Review

Our protocol was drafted using the Preferred Reporting Items for Systematic Reviews and Meta-analysis Protocols (PRISMA-P). The following databases were searched: PubMed/MEDLINE (October 2020–April 2021), Web of Science (October 2020–April 2021) and Worldcat (October 2020–April 2021). We also searched for literature using the Google search engine and the first 20 results were reviewed as this search engine displays the results by relevance. Articles that were published and peer reviewed, as well as official documentation by the Mental Health Commission of Canada, were examined in this study. The reference lists of articles selected for full-text review were searched for additional articles. We also used Google Scholar to identify other published scholarly literature by performing a title and author searches. All publication/study types and languages were included in this search strategy. There were no limits placed and no restriction on the year of publication, with databases searched back to their inception. Search terms used in Google/online databases to find the article include: “coronavirus,” “COVID-19,” “mental health,” “anxiety,” “depression,” “isolation,” “mental health resources,” “peer support,” “online mental health resources,” and “healthcare workers.” Example of search query used in PubMed database: [(“coronavirus” OR “COVID-19”) AND (“mental health” OR “anxiety” OR “depression” OR “isolation”)].

### The Prevalence of Poor Mental Health During the COVID-19 Pandemic

Worsening mental health has become an increasingly concerning issue in countries instituting measures such as mandated lockdowns. Recent survey studies done revealed that large proportions of the sampled population showed a higher prevalence of stress, anxiety, and depression as a result of lockdowns and physical distancing (9). For example, in Italy, the period of the first lockdown had significant detrimental effects on the mental health of its citizens (10). In fact, one survey administered in Italy revealed that the perceived levels of happiness and mental health decreased as a result of the lockdown, while their feelings of loneliness increased (11). Other studies have shown that the negative psychological effects experienced by adults also included confusion, anger, and distress (3). There are several stressors experienced during quarantine which can lead to these negative affective states such as the duration of quarantine, fears of infection, frustration, boredom, insufficient supplies, and inadequate information (12). Additionally, the constant fears of the unknown, stigma, and state of one's finances can continue to act as stressors post-quarantine (12).

Amongst children 11-years of age or younger, and youth 18- to 24-years of age, a significant portion reported experiencing increased loneliness during the pandemic due to lockdowns, social distancing, and school closures, with the latter age group being affected the most (3, 10). The loneliness was associated with depressive symptoms, social isolation, generalized anxiety, suicidal ideation, self-harm, and eating disorder behavior (3). One study which analyzed parent-reported levels of mental well-being of their children isolated in the context of various infections revealed increased levels of adjustment disorder, acute stress, grief, and post-traumatic stress disorder (PTSD). One reason for this could be the important role that peer groups and social identity plays in the development of young individuals (13, 14). This is an issue of great concern as politicians and policymakers in various countries determine the length of instituted lockdowns, school closures, and social distancing in the context of COVID-19.

### The Impact of the Pandemic on the Mental Health of Healthcare Workers

One portion of the population that is severely impacted by this pandemic are the frontline medical workers. In fact, surveys done in China, Togo and India during this pandemic have shown that there is a higher prevalence of insomnia, anxiety, depression, somatization, and obsessive-compulsive symptoms in medical workers compared to non-medical workers (15–17). One study that interviewed hospital staff who were quarantined during the first SARS outbreak found that they were more likely to report feeling exhaustion, detachment from others, irritation, poor sleep, poor concentration, and ultimately a decline in their performance in the workplace (18). In fact, survey-based analysis done on healthcare workers during the initial COVID-19 outbreak in China revealed that 23.2% of them experienced anxiety, 22.8% showed prevalence of depression, and 38.9% experienced insomnia (19). Moreover, there were sex-specific differences with female healthcare workers experiencing more of the affective symptoms than their male counterparts (19).

These are extraordinarily difficult times for those working in the inpatient units, consul liaison services, emergency departments, as well as those having to widen their scope of practice in the presence of an overloaded healthcare system (20). This underscores the need for an accessible support system to aid those that directly provide care at the frontlines during the COVID-19 pandemic. To address this decline in healthcare worker mental health, it has been suggested that there be increased provisions of mental health support which will result in greater self-efficacy and confidence (21). Additionally, the use of technology to deliver psychosocial supports while preserving social distancing would be greatly beneficial (21).

### The Lack of Accessible and Effective Mental Health Resources During the COVID-19 Pandemic

Social distancing and lockdown measures have forced many individuals to stay inside, leaving them unable to access traditional mental health services. Moreover, many support services are not able to effectively transition to an online delivery model in an accessible manner. To address the issue of dwindling mental health, many governments are making the effort to advertise resources such as telephone helplines, videos, and readable material (22, 23). Though they can serve to inform, they are often inadequate in meeting the increased need of mental health resources caused by the COVID-19 pandemic (24).

Many digital interventions currently exist to address social isolation and loneliness in young people. Computerized cognitive-behavioral therapies such as BRAVE-TA, MoodGym, SPARX, and “Think, Feel, Do” have small, but positive impacts on mental health (25–27). Additionally, self-help interventions such as bibliotherapy and computerized therapy have shown to have a somewhat positive effect on mental health but are agreed to generally be less effective than face to face therapies (28–30). Unfortunately, even though mobile apps are a very accessible and easy-to-use medium of obtaining information and accessing resources, there is a lack of evidence supporting their effectiveness in improving mental health (25, 31, 32).

Fortunately, for healthcare workers, an evidence-based community forum known as *Schwartz Rounds* allows medical staff from all backgrounds to discuss their emotions regarding work-related matters (33). Moreover, those who used this online forum reported feeling acknowledged and validated (33). Nonetheless, staff are still encouraged to use institutional peer support programs as a single forum-based resource may not be sufficient (34).

In order to make mental health resources more accessible, platforms such as WeChat, Weibo, and WhatsApp are being used in many countries to better connect individuals with psychological counseling services (23, 35). Yet, the risk of increasing suicide mortality amongst regular citizens and health care workers is of great concern given the present economic stress, social isolation, decreased access to community and religious support, and barriers to mental health treatment and illness (36). Hence, it has been suggested that having a telephone support line that is staffed by nurses and/or counselors would be beneficial for individuals in quarantine (37).

Community is sought during times of strife and the differences in access to adequate mental health support across different socioeconomic groups makes it challenging to find a reliable resource (20). This suggests the need for an accessible form of support that can remove barriers between a struggling individual and a supporter. There is currently evidence for the utility of telepsychiatry in aiding the mental health of growing adults and adolescents (38). However, the limitations of this approach include the need for trained psychiatric counselors, as well as the stigma associated with seeking professional help (39).

### Peer Support as a Viable Mental Health Resource

Peer support as a mental health resource has grown exponentially in the last few decades around the world, namely North America and Europe (40). In the US alone, Goldstrom et al. has reported that services run by, and delivered to, people with mental health issues more than double traditional, professional mental health organizations (41). This growth is supported by numerous studies that illustrate the safety and efficacy of peer support which include its ability to improve empowerment, hope, quality of life, self-esteem, social functioning, and care engagement for those accessing its services (40, 42–45). Furthermore, two comprehensive systematic reviews have shown that not only are peer supporters able to achieve similar outcomes to mental health professionals, but that peer supporters reduced inpatient service use and improved relationships with providers, care engagement, and various recovery-related outcomes in people struggling with severe mental illness when compared to professionals (46, 47). On the other hand, one of the systematic reviews did identify a study that found that the presence of a peer increased the number of psychiatric hospitalization days (46). They postulate this could be because the peer heightened the awareness of the clients' suffering and accordingly advocated for interventions leading to additional hospitalizations (48). In the other studies with the positive results, however, the recipients may have felt that the peer support provided was sufficient and that further help in the form of professional services was not required. Overall, there are many peer support services and organizations that are used to aid the mental health of patients with various mental and physical ailments, but the general consensus toward peer support is that it is either inconclusive or yields positive effects (Table 1). Notably, the presence of conflicting results reiterates the need for further research to better characterize the impact of peer support.

**Table 1:** Summary of peer support resources investigated in peer-reviewed literature.

**Type of peer support administered**	**Type of participants**	**Number of participants**	**Type of study**	**Outcome**	**Reference**
Mutual self-help groups led by professionals/clinicians and consumer-led programs	People diagnosed with schizophrenia or other related serious mental illness	13 studies, 2,479 participants	Systematic review	Inconclusive evidence on impact of peer support on individuals with schizophrenia.	(49)
Community-based peer and community health worker-led diabetes self-management programs (COMP-DSMPs)	Adults with either type 1 or type 2 diabetes	11 studies, 6,090 participants	Systematic review	Peer support programs show limited, inconsistent benefits for adults with diabetes in low- and middle-income countries.	(50)
Dyads (one-on-one), groups (group-based peer education), and combination	Adults with either type 1 or type 2 diabetes	25 RCTs, 8,942 participants,	Systematic review	Mixed evidence for facilitation of changes in health-related behaviors.	(51)
Majority group face-to-face, peers received some training	Adults with diabetes	25 studies, 4,800 participants	Systematic review	Inconsistent improvement in health factors in adults with diabetes.	(52)
Wide variety of programs targeting complex health behavior change, including addiction, cardiovascular disease, diabetes, HIV/AIDS, maternal and child health, mental health and other chronic disease	Adults with various complex health concerns	65 studies	Systematic review	Significant evidence for peer support improving complex health behaviors in disease prevention and management.	(53)
Interventions that placed individuals with current depression in regular contact with at least one other person with either current or prior depression	Adults with current symptoms of depression	7 RCTs, 869 participants	Meta-analysis	Peer support interventions help reduce symptoms of depression.	(54)
Peers working in statutory of professionally led services offering support to people with mental health problems	Adults with various mental health problems	7 RCTSs	Systematic review	Peer support workers can reduce admissions among those with whom they work and have a positive impact on the lives of people with mental health problems.	(40)
Peer support telephone calls	Adults with various health concerns	7 RCTs, 2,492 participants	Systematic Review	Peer support telephone calls can be effective for certain health-related concerns.	(55)
Various volunteer delivered programs	Adults with cancer	17 programs	Systematic review	Peer support provides benefits to cancer patients.	(56)
Family, friends and significant others	College students	239 college students (117 females)	Structural equation modeling, survey design	Stress and social support affect adjustment; social support is more important for females, coping behaviors for males.	(57)
Friends and family	College students	75 college students (54 females); undergraduate education degree; 22–48 y/o, 29 males	Empirical study, survey design	Social support correlates with emotion-focused coping strategies; females report more social support from friends.	(58)
Friends, family, co-workers, community members, etc.	College students	531 college students (51% female); 80% White, 13% Asian/ Pacific-Islander, 7% African-American, Latino/ Mexican-American, other	Empirical study, survey design	Females were more anxious, especially those with less social support; anxiety of males was unrelated to social support.	(59)
Family, friends and significant others	College students	2,843 college students	Empirical study, web-based survey	Students with lower quality support were more likely to experience mental health problems.	(60)
Emotional, informational and tangible support, and satisfaction with support	College students	439 undergrad students (71% females)	Cross-sectional, survey design	Positive social support, particularly tangible support, and negative social exchange were significantly predictive of greater suicidal behavior.	(61)
A student-led service that provides peer support	College students	1,093 university students and 797 volunteers	Empirical study, survey design	A service that provides peer support is beneficial to the members of a university/college campus.	(62)
Family, friends and significant others	College students	180 college students (122 females); 18-46 y/o, 21 males; 94% Caucasian; 51% 1st-year, 23% 2nd-year	Prospective empirical study, survey design	Loneliness was predicted by reductions in close social support, especially among those who were very shy.	(63)
Parents and friends	College students	197 1st and 2nd-year undergraduate students at an urban private university (61% females); 93% 18–19 y/o; 77% White, 11% Asian, 7% Hispanic, 1% African-American, 4% Other	Quantitative empirical study, survey design	Depression and self-esteem were significantly negatively correlated with peer support and student-reported parental support.	(64)
Friends and family	College students	101 1st-year college students (65 females); 17–19 y/o, 18 males; 25% White, 18% Japanese, 15% Mixed-race, 12% Hawaiian, 12% Chinese, 10% Filipino, 4% Korean, 14% Other	Quantitative empirical study, survey design	Support from family and friends positively impacts the commitment to the goal of graduation and their intention to persist.	(65)
Friends and family	College students	214 undergraduate students (148 females); small liberal arts university; mainly 1st and 2nd-year	Empirical study, survey design	Females have more social support; burnout in females related to Personal Accomplishment (indicates wrong type of social support); burnout in males related to Depersonalization (indicates lack of emotionally supportive contacts).	(66)
Parents and best friends	College students	272 college students (66% female); 90% White	Quantitative empirical study, survey design	Social support, social competence and social connectedness are strongly related to psychological health.	(67)
Close friends, family members, classmates, professors and other people at the school	College students	304 community college students (78% female); 75% White; 5 males; 77% working; 36 with children; 75% 1st generation.	Quantitative empirical study, survey design	Social support is related to academic persistence, buffer negative effects of stress	(68)
Social support intervention carried out by lay non-professional volunteers; friendly visiting	Older adults, mean age = 83	80 participants	RCT	Social support improves frailty status in prefrail and frail community-dwelling older persons.	(69)
Paraprofessional women employed to deliver social support services through home visiting, received 3 weeks of intensive training	Girls 18 years of age bearing their first child	Treatment = 1,901Control = 4,613	Quantitative empirical study	Social support program decreases preterm birth in teenage mothers.	(70)
Individual and family counselling sessions, then weekly support groups, opportunity for *ad hoc* consultation	Primary caregiver married to and still living with a patient who had received a clinical diagnosis of Alzheimer's disease	Treatment = 103Control = 103	RCT, survey design	Social support program prevented depressive symptoms in spouse-caregivers of Alzheimer's patients.	(71)
Looked at social indicators, specifically value of giving social support	Citizens of 23 European countries (ages 15–103)	44,238 respondents	Quantitative empirical study, survey design	Community social support may have a protective effect against suicide, especially for males and for individuals in high suicide rate regions.	(72)
Peer support from trained volunteer survivors	Participants in the suicide bereavement peer support program	19 participants	Mixed-methods evaluation	Unsupervised peer support provided positive short-term outcomes to bereaved participants.	(73)
Year-long therapeutic intervention program, including primarily social support	Inmates with a narcotics addiction	Social treatment = 50Social + spiritual treatment = 43	RCT	Peer support improved depression and hostility in recovering addicts in prison.	(74)
An older adult volunteer is paired with a participant (an older adult who is to receive peer support); volunteers are trained	Older adults who received Medicaid	32 participants	Empirical study, survey design	Peer support alleviates depression but not anxiety symptoms in older adults.	(75)
Peer-moderated support group led by two trained peer-facilitators, with medical advisor present	Women suffering from post-partum depression	Participants = 118Control = 152	Empirical study, survey design	Peer support for women suffering post-partum depression provides a slight improvement in depressive symptoms.	(76)
Peer-to-peer phone support prevention program	Parents of at-risk youth with significant emotional and behavioral difficulties	139 participants	Empirical study, survey design	Peer support program for parents or at-risk youth improved perceived social and concrete support.	(77)

*RCT, randomized control trials*.

Looking specifically at educational institutions, peer support has been shown to improve self-esteem, anxiety, depression, stress, burnout, loneliness, and overall mental well-being, although the literature here is limited (Table 1) (78–81). Interestingly, one of these studies determined that structured peer support is unlikely to have a significant effect on improving early and preventative intervention (79). Although they expected peer support to facilitate early intervention for students reluctant to access professional services, they found that many of the students attending peer support groups had already sought professional support and had been experiencing mental health difficulties for over a year. Therefore, they argue that integrating peer support into professional-led services may maximize outcomes as opposed to pursuing one intervention over the other (79).

However, peer support can pose a few challenges. Previous studies have demonstrated concerns regarding boundaries and power dynamics but also the stress, accountability, and risk assessment peer supporters are faced with in their roles, and so it is imperative that they prioritize their own mental health as well (40, 82–85). One systematic review has found that there was little to no evidence that peer support impacted hospitalization or overall symptoms, and that the positive effects reported on hope, recovery, and empowerment were present but inconsistent (86). Altogether, they state that current evidence is promising but does not support the requirement of mental health services to provide peer support programmes (86). One reason for this could potentially be because peer support is a relatively new phenomenon in the mental health landscape, that is only recently getting traction for being a viable mental health resource. Therefore, additional research studies and clinical trials are required to better understand the role of complex interventions such as peer support.

### Strategies for Obtaining and Providing Peer Support in the Context of the COVID-19 Pandemic

Another strength of peer support is its flexibility. Peer support can be provided in various settings through several different mediums as illustrated in Table 1. The first distinction is whether the peer support is provided in a group or an individual setting. Group peer support typically functions with a peer support facilitator and then multiple service users who each share and discuss their experiences with the group. Perhaps the best and oldest example of group peer support, and peer support in general, is Alcoholics Anonymous (AA) (87). AA is an established and heavily researched peer support delivery method with a recent review indicating social support variables such as shared experiences as being a key factor to its effectiveness (87). Group peer support methods have now been successfully deployed in various settings, ranging from mental health focused groups such as the Wellness Recovery Action Plan (WRAP) (88) and Building Recovery of Individual Dreams and Goals through Education and Support (BRIDGES) (89) programmes to supporting HIV-infected adolescents (90) and improving quality of life in breast cancer patients (91).

Individual peer support, on the other hand, is typically a one-on-one delivery with a single supporter and service user (88–91). These interactions have been shown to provide beneficial practical, emotional, and social supports in a non-treatment based, normalizing relationship but are highly understudied and lack evidence regarding the necessary duration, frequency, quality, or intensity to maximize its effectiveness (92). For these reasons, peer support has been acknowledged as a resource that should complement rather than replace professional mental health resources at this time, suggesting the fact that a blended approach may work synergistically to maximize mental health outcomes (79). The benefit of professional mental health services is not to be understated as they indeed have been proven to be very useful for aiding one's mental health, however, numerous barriers can still prevent individuals from obtaining the help they need (93–95). Therefore, alternative sources of support need to be explored, and notably, individual peer support is becoming an extremely common delivery method that continues to grow in popularity (40, 41).

With the arrival of the COVID-19 pandemic and the swift transition of many services to online platforms, virtual peer support services have quickly come to the forefront of novel mental health support delivery methods. By removing traditional barriers to peer support programs such as accessibility and availability, virtual peer support may be a very promising alternative to in-person peer support *via* utilization of video conferencing software such as Zoom, Google Meets, and Cisco Webex. A 2019 article by Fortuna et al. summarizes the diverse technology modalities that digital peer support can be delivered through, which includes “peer-delivered and smartphone-supported interventions, peer-supported asynchronous technology, artificial peer support, informal peer-to-peer support *via* social media, video games, and virtual worlds” (96). Unfortunately, research on virtual peer support is limited with various systematic reviews identifying an overall lack of high-quality studies in online peer support, mainly because many of these interventions are used adjunctively and therefore the individual effect of online peer support cannot be clearly demonstrated (97–100). Nonetheless, the findings they do note are promising and could be the next frontier of peer support and mental health care in the future (97–100).

Collectively, through reviewing peer-reviewed studies, we identified the following steps as being effective ways to provide peer support during the COVID-19 pandemic:

**In person (for the people within your social-distancing bubble):**

1. Find a comfortable and welcoming room/space with minimal interruptions.
2. Reassure the person you will be confidential, non-judgemental, and non-directional toward anything that they will say.
3. Actively listen using minimal encouragers and appropriate body language. Additionally, validate and normalize the individual's thoughts, feelings, and experiences. Note: the supporter should try and maintain a balance between listening 80% of the time and talking 20% of the time.
  - Some example phrases/sentence starters to use:
    ○ “From what I am hearing, it sounds like…”
    ○ “It is understandable why someone in your position would feel like that.”
    ○ “Thank you for opening up to me, it takes a lot of courage to share something so personal.”
    ○ “Your experience is unique, but anyone in a similar position would feel that way.”
    ○ “You're not alone.”
4. Paraphrase and summarize the key points of what they are sharing with you. Ask for clarification and allow them to correct you as necessary.
5. Ask open-ended question to encourage conversation and keep it flowing organically.
  - Some example open-ended questions to ask:
    ○ “How did you feel before that happened? How did you feel after?”
    ○ “How has this affected your sleep, eating habits, etc.”
    ○ “What do you like to do for self-care?”
    ○ “How can I offer you support? Please tell me what that looks like to you.”
6. If the individual is seeking advice or counsel, you may brainstorm potential ideas with them non-directionally, but have them lead the discussion.
  - This can be done through redirection:
    ○ “I do not know your experience as well as you do. What do you feel you should do? Why?”
    ○ “Before I share any of my thoughts, I would love to hear a bit more about what things you have already considered.”

**Virtually (for the people outside of your bubble):**

1. Find a comfortable room or space with an appropriate/welcoming background and minimal noise.
  - Note: using earphones is highly encouraged if there are other people passing by in that area.
2. Open a video conferencing software such as Google Meets, Zoom, WebEx, etc. on a computer or mobile device and send the individual the meeting link.
3. Allow them to keep the video on/off depending on what they prefer and assure them that they are free to leave the call at any time.
4. Reassure the person that you will be confidential, non-judgemental, and non-directional toward anything that they will say.
5. Actively listen using minimal encouragers and appropriate body language. Additionally, validate and normalize the individual's thoughts, feelings, and experiences. Note: the supporter should try and maintain a balance between listening 80% of the time and talking 20% of the time.
  - Some example phrases/sentence starters to use:
    ○ “From what I am hearing, it sounds like…”
    ○ “It is understandable why someone in your position would feel like that.”
    ○ “Thank you for opening up to me, it takes a lot of courage to share something so personal.”
    ○ “Your experience is unique, but anyone in a similar position would feel the same way.”
    ○ “You're not alone.”
6. Paraphrase and summarize the key points of what they are sharing with you. Ask for clarification and allow them to correct you as necessary.
7. Ask open-ended question to encourage conversation and keep it flowing organically.
  - Some example open-ended questions to ask:
    ○ “How did you feel before that happened? How did you feel after?”
    ○ “How has this affected your sleep, eating habits, etc.”
    ○ “What do you like to do for self-care?”
    ○ “How can I offer you support? Please tell me what that looks like to you.”
8. If the individual is seeking advice or counsel, you may brainstorm potential ideas with them non-directionally, but have them lead the discussion.
  - This can be done through redirection:
    ○ “I do not know your experience as well as you do. What do you feel you should do? Why?”
    ○ “Before I share any of my thoughts, I would love to hear a bit more about what things you have already considered.”

- **Troubleshooting tips for virtual peer support:**
  - If the video call lags, be honest about it and ask them to repeat what they had said.
  - If the call ends unexpectedly, send them another meeting link and continue from where you left-off.
  - If the call is lagging excessively, turning off the video may reduce the lag.

## Concluding Remarks

The COVID-19 pandemic has had devastating effects on communities all around the world. Notably, its impact on the mental health of individuals by way of instituted lockdowns and social distancing/isolation measures remains to be sufficiently addressed. Although professional mental health services have begun transitioning to online delivery models, this adaptation has not been sufficient to meet the growing need of mental health support. That being said, there still remains viable options for those in need. Peer support, although not a psychiatric or professional service, can be generally beneficial in improving mental health. This review identifies numerous studies illustrating the positive impacts of peer support, but the presence of studies demonstrating its lack of utility should not be ignored. It should be noted that the vast majority of the peer support studies discussed here have not identified any clear adverse outcomes on recipients' mental health and so, it appears that peer support can actually be a quite safe and beneficial resource. More research is necessary, but for the time being, utilizing peer support as a complementary resource to professional services may maximize positive outcomes. During the pandemic, however, peer support stands out as a resource that can be easily provided by members of the community to one another through accessible online mediums, even during times of quarantine and lockdown. As this pandemic draws on for extended periods of time, mental health can deteriorate. However, members of the community can help one another through the provision of peer support to ensure that all members of society are able to endure these trying times.

## Author Contributions

RS: conception of review topic, writing of review, table analyzing peer-reviewed literature on peer support, and editing of manuscript. AA: writing of review and editing of manuscript. ZK: table analyzing peer-reviewed literature on peer support. All authors contributed to the article and approved the submitted version.

## Conflict of Interest

The authors declare that the research was conducted in the absence of any commercial or financial relationships that could be construed as a potential conflict of interest.

## Abbreviations

COVID-19: coronavirus-19
PTSD: post-traumatic stress disorder
RCT: randomized controlled trial.
